# Can a precast pit latrine concrete floor withstand emptying operations? An investigation from Malawi

**DOI:** 10.2166/washdev.2018.096

**Published:** 2018-12-07

**Authors:** Joshua Mchenga, Rochelle H. Holm

**Affiliations:** **Joshua Mchenga, Rochelle H. Holm** (corresponding author) Centre of Excellence in Water and Sanitation, Mzuzu University, P/Bag 201, Luwinga, Mzuzu 2, Malawi

**Keywords:** concrete, fecal sludge management, Malawi, pit latrine, safety, sanitation

## Abstract

For fecal sludge from households in low- and middle-income countries to be treated offsite it needs to be removed, which can be greatly affected by the pit latrine floor design. However, it is unclear whether precast pit latrine concrete floors (latrine slabs) can withstand emptiers and their equipment. To investigate this issue, 28 prefabricated latrine slabs were purchased in two cities of Malawi. They were first visually evaluated, and then their compression strength was tested. Additionally, each seller was asked a series of questions to better understand their business, training, and construction practices. Results showed that households should perform due diligence to ensure that they are purchasing a safe precast latrine slab. Commonly reported problems included nonstandard reinforcement material and spacing, in addition to slabs that were not thick enough or were not large enough in diameter. The results of this research illustrate the inherent complexity in ensuring high-quality decentralized sanitation solutionsand how one component, the user interface, if implemented poorly, can affect the rest of the value chain. The findings from this work can help inform training and initiatives that engage artisans and suppliers who play a role in the provision of onsite sanitation service delivery.

## INTRODUCTION

Many households in low- and middle-income countries currently use and will continue to use pit latrines, the contents of which need to be treated either onsite or offsite. The purpose of the pit latrine floor is to cover the pit, effectively separating and containing the fecal sludge while also supporting self and imposed load; this structure is the first checkpoint in the sanitation value chain. The most common urban household sanitation facility in Malawi is a pit latrine with a concrete floor (latrine slab). Most of these facilities are shared by several families (National Statistical Office & ICF 2017). Typically, pit latrine concrete floors are constructed in one of two ways: a slab is poured onsite over the top of the pit, or a slab is prefabricated and installed. Prefabrication of concrete pit latrine slabs is becoming a popular way to encourage private sector sanitation service providers selling these products and to allow faster construction during emergencies (Harvey 2007; WEDC 2012; Centre for Affordable Water and Sanitation Technology (CAWST) 2014; Holm *et al*. 2018).

For fecal sludge to be treated offsite, it needs to be removed from the pit. This process can be affected greatly if the pit latrine floor was not constructed correctly. Researchers estimate a typical adult mass of 61 kg (Walpole *et al*. 2012). The pedal powered modified Gulper pump, developed locally at Mzuzu University for pit latrine emptying, is 59 kg and accesses the pit through a hole in the latrine slab (the squat hole). It requires two operators for use (Chipeta *et al*. 2017). Consequently, a pit latrine floor inMalawi must be able to hold up to 200 kg during emptying operations. Common latrine slab designs in Malawi are either a concrete flat square or a circular dome. Despite the presence of a concrete slab, the below ground surface pit may not have been lined, and an earth floor is likely to be even more unsafe for emptying operations.

Concrete is not very flexible. Variability in precast latrine slab construction is difficult to observe, whereas for slabs cast in situ quality standards during construction may be observed directly. Although neighboring Zambia has basic latrine construction regulations (Zambia Government 1994), Malawi has only a National Sanitation Policy (Malawi Government 2008) but no national regulations for pit latrine construction.

With latrine slab businesses becoming more prevalent but severely under-researched, this case study explores the variability in precast slabs sold by private sector sanitation service providers and whether they can withstand the mass of two adults and their emptying equipment.

## METHODS

This study took place in the cities of Lilongwe and Mzuzu, Malawi. Purposive sampling was used to purchase precast pit latrine concrete slabs marketed for household use (Lilongwe *n* = 20 and Mzuzu *n* = 8), from different geographic areas in each city and from different sellers. The term ‘seller’ is used to refer to the private sector sanitation service provider from whom the latrine slab was purchased for this study and may include masons or distributors, for example. In Lilongwe, the 20 slabs were purchased from ten sellers. In Mzuzu, the eight slabs were purchased from five sellers. This study did not consider sanitation platforms (SanPlat).

A face-to-face interview guide with the seller on the day of purchase was used, covering details including the number of years in business, previous training in slab production, the concrete mixture used, and the presence, size, and placement details of reinforcement materials. Researchers also documented the cost of the slab purchased. Interviews were conducted in the local language, Chichewa. An exchange rate of Malawi Kwacha (MK)720 = USD$1 was used.

For each slab (*n* = 28), researchers completed a check-list based on visual observations, including the dimensions and the presence of cracks, disintegration, lifting inserts, or ventilation holes.

**Figure 1:**
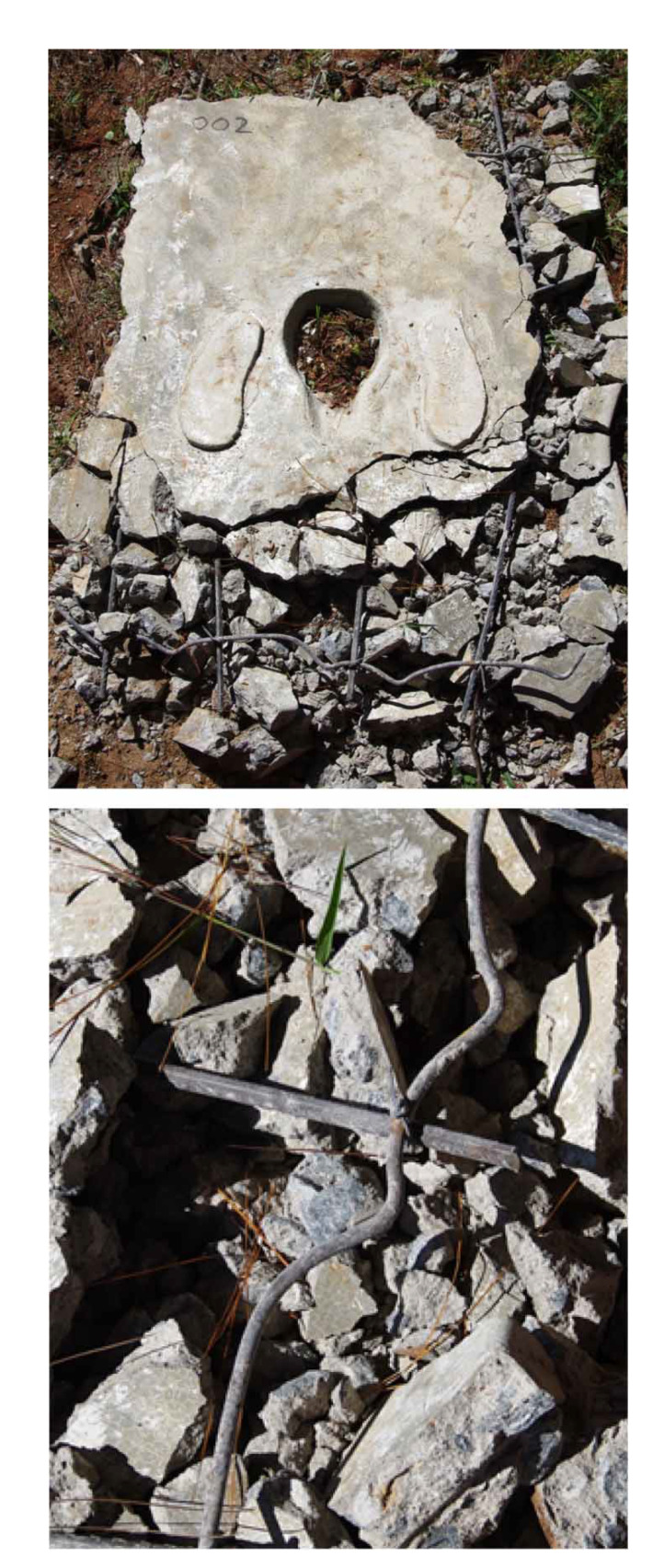
Precast pit latrine flat slab. Top: reinforcement bar verification exercise. Bottom: reinforcement bar using recycled material

To test the ability of each slab to hold the mass of two emptiers and their equipment, the rebound hammer test was selected. The rebound hammer is a nondestructive test used to determine compressive strength as an indicator of concrete hardness and quality, but cannot estimate the strength of the concrete (Malhotra & Carino 2004; Szilágyi & Borosnyói 2009). For this work, the rebound hammer (Proceq type N-34, model number 111510) was calibrated prior to the activity and was used by laboratory personnel from the Ministry of Transport and Public Works, Malawi. Ten readings using the same hammer were performed for each slab in the vertically downward position. Thereafter, a reinforcement material verification exercise was conducted by manually crushing the slab with a sledgehammer (Figure 1). The size and spacing of the reinforcement were recorded as were the materials used in their construction and whether the reinforcement materials were corroded. The results were compared to technical specifications by Harvey (2007) and WEDC (2012).

Ethical approval for this research was obtained from the Malawi Government, National Commission for Science and Technology (study protocol number P02/18/255).

### RESULTS AND DISCUSSION

The results of this study provide guidance in terms of what options should be recommended to improve household sanitation facilities in low- and middle-income countries. These facilities ultimately require fecal sludge to be treated offsite while there is also an important role promoting private sector sanitation service providers selling these products.

In Lilongwe, no sellers had dome slabs; only three dome slabs could be found in Mzuzu.

#### Slab dimensions

Earlier work in Mzuzu found a mean pit diameter below ground surface of 0.8 m (Chiposa et al. 2017). Considering the recommended minimum 0.05 m overlap (the part of the slab that rests on the top of the pit), slabs should be at least 0.9 m in length and width or for dome slabs in diameter. In this study, it was found that most (20/25) flat slabs passed these recommended dimensions; however, none of the dome slabs met the 0.9 m diameter requirements. Many (19/25) flat slabs met the 0.065 m thickness minimum, with a few (7/19) more than 0.08 m thick. Although each dome slab (3/3) was at least 0.04 m thick, none had the recommended dome height of 0.1 m at the center. Each (28/28) slab had a keyhole-shaped squat hole for waste dispo-sal. Most (24/28) slabs featured a squat hole that was placed correctly, at least 0.25 m from the back. However, fewer (15/ 28) slabs had squat holes of the recommended dimensions, a width of 0.16-0.18 m in diameter and 0.25-0.4 m long.

Only three of the slabs (3/28) had a cutout for a ventilation pipe.

#### Presence of reinforcement

Each seller indicated during their interview that their slab (28/28) contained reinforcement. None of the slabs had reinforcement exposed or not fully covered with concrete at the time of purchase.

For each of the dome slabs (3/3), the material (binding wire) reported by the seller was observed to be accurate during the reinforcement verification exercise. However, the dome slab, if constructed properly, does not require any reinforcement, and the cost of dome slab construction could be reduced and the seller profit possibly increased if such reinforcement is not used.

While all of the flat slabs (25/25) had some form of reinforcement, it was observed to be of variable material, size, and spacing (Table 1). Notably, instead of steel reinfor-cing bars, four slabs had used scrap metal (such as reusing a window frame of a car and a sofa spring), and some of this scrap material was observed to be corroded (Figure 1). Two other flat slabs only used binding wire for reinforcement. In one case, the slab mostly had the proper size of reinforce-ment, but one length of reinforcement was substituted with a sofa spring. None of the flat slabs met the reinforcement recommended by Harvey (2007).

**Table 1:** | Summary results of individual precast pit latrine concrete slabs (n ¼ 28)

Slab ID	City	Slab type	Price (Malawi Kwacha)	Did seller report receiving training?	Slab length/ diameter (m)	Slab width (m)	Slab thickness (m)	Are surfaces free from visual cracks, chipping or unevenness?	Compression strength (N/mm2)	Reinforcement material observed
1	Mzuzu	Flat	25,000	Yes	1.41	1.01	0.05	Yes	<20	Reinforcing bar
2	Mzuzu	Flat	25,000	Yes	1.40	1.00	0.05	Yes	<20	Reinforcing bar
3	Mzuzu	Flat	14,000	Yes	0.66	0.52	0.07	No	<20	Reinforcing bar
4	Mzuzu	Flat	14,000	Yes	0.66	0.53	0.06	No	<20	Reinforcing bar
5	Mzuzu	Flat	15,000	Yes	1.32	0.69	0.06	Yes	<20	Reinforcing bar and scrap metal
6	Mzuzu	Dome	15,000	Yes	0.81		0.07	Yes	<20	Wire
7	Mzuzu	Dome	15,000	Yes	0.80		0.08	Yes	<20	Wire
8	Mzuzu	Dome	10,000	No	0.63		0.07	No	<20	Wire
9	Lilongwe	Flat	18,000	No	1.30	0.90	0.07	Yes	<20	Reinforcing bar and scrap metal
10	Lilongwe	Flat	18,000	No	1.30	0.91	0.06	Yes	<20	Reinforcing bar and scrap metal
11	Lilongwe	Flat	17,500	No	1.05	0.80	0.09	Yes	<20	Reinforcing bar
12	Lilongwe	Flat	17,500	No	1.30	0.90	0.07	Yes	<20	Reinforcing bar
13	Lilongwe	Flat	15,000	No	1.01	0.91	0.08	No	<20	Reinforcing bar
14	Lilongwe	Flat	15,000	No	1.01	0.91	0.08	No	<20	Reinforcing bar
15	Lilongwe	Flat	15,000	No	1.01	0.91	0.08	No (minor cracks present)	<20	Reinforcing bar
16	Lilongwe	Flat	15,000	No	1.20	0.90	0.07	No (minor cracks present)	<20	Reinforcing bar
17	Lilongwe	Flat	16,000	Yes	1.01	0.90	0.07	Yes	<20	Reinforcing bar
18	Lilongwe	Flat	16,000	Yes	1.32	0.90	0.08	Yes	25	Reinforcing bar
19	Lilongwe	Flat	17,500	No	1.32	0.90	0.08	Yes	<20	Reinforcing bar
20	Lilongwe	Flat	17,500	No	1.20	1.00	0.07	Yes	<20	Reinforcing bar
21	Lilongwe	Flat	16,000	No	1.26	0.90	0.08	Yes	<20	Reinforcing bar
22	Lilongwe	Flat	16,000	No	0.91	0.91	0.08	Yes	<20	Scrap metal
23	Lilongwe	Flat	16,000	No	1.25	0.90	0.07	Yes	<20	Reinforcing bar
24	Lilongwe	Flat	16,000	No	1.27	0.91	0.07	Yes	<20	Reinforcing bar
25	Lilongwe	Flat	15,000	No	1.17	0.90	0.07	Yes	<20	Wire
26	Lilongwe	Flat	15,000	No	1.40	0.90	0.07	Yes	<20	Wire
27	Lilongwe	Flat	16,000	Yes	0.91	0.81	0.07	Yes	<20	Reinforcing bar
28	Lilongwe	Flat	16,000	Yes	1.20	0.90	0.05	Yes	<20	Reinforcing bar

#### Compressive strength

The compression strength between slab samples is a strong indicator of durability. Each seller (28/28) reported that the slabs had been cast at least 21 days prior to data collection. Each of the slab surfaces was troweled. Only one slab had a compression strength >20 N/mm^2^, the minimum applicable curve for the rebound hammer used in this study, which is an indication that the recommended concrete mix proportions were used. When the researchers constructed a flat slab based on the quality standards of Harvey (2007) and WEDC (2012), the compressive strength was found to be >20 N/mm^2^, which indicates that if constructed properly, the slabs in this study should be able to achieve this threshold compressive strength.

For slab construction, a ratio of cement to sand to gravel of 1:2:4 or 1:3:6 is recommended (Harvey 2007). Some sellers did not know the ratios used in construction, but when available it was reported that the ratios ranged from 1:5:1 to 1:1:2. No seller reported the use of either of the recommended ratios.

#### Cost

Many independent variables affect the selling price of slabs, including taxes, labor, and transportation (whether the seller has their own transport to collect materials or uses public transport). The current minimum wage in Malawi is MK25,000 (USD$35) per month. When researchers constructed a flat slab based on the quality standards of Harvey (2007) and WEDC (2012), the cost was MK26,311 (USD$37) (materials MK15,630; transport MK8,300; 2 days labor MK2,381). The cost of each slab in this study was even lower. A provider who builds slabs that meet quality standards may not be able to compete with the prices of inferior but aesthetically pleasing products on the market.

#### Seller businesses

It was more common for sellers to have received formal training in slab construction in Mzuzu (4/5 sellers) than in Lilongwe (2/10 sellers). The duration of training was reported to be 1 week and was mostly overseen by nongo-vernmental organizations. The reported slab production per seller was 2 to 20 slabs per week, indicating a relatively small-scale business. Many sellers (10/15) had been in business for more than three years.

It was observed by researchers in this study that the delivery of precast latrine slabs from the seller to the household is challenging and expensive (MK2,000 to load the slabs into the buyer's vehicle and an extra MK5,000 to transport the slabs). The use of local public transport for a slab is difficult; for example, consider the task of lifting a slab onto the top rack of a local minibus. Only two (of 28) slabs had lifting inserts.

Rahman et al. (2013) reported that in Malaysia, prefabricated toilet construction allows promotion by sellers of proprietary designs, cost savings through mass factory production, and testing in advance under a controlled factory environment; however, none of these factors were observed among the sellers in this study.

#### Study limitations

It was not possible to determine the exact mixture or age of the concrete, but researchers estimated by visual observation that the studied slabs were all older than 28 days. The rebound hammer was not as useful as planned due to the generally low compressive strength values of the slabs. Destructive test methods were not available for this study and are recommended for future research. The sample size in Mzuzu was limited by the available slabs on the market, but this may be typical of a smaller urban area with few private sector sanitation service providers. Furthermore, the study had a limited sample size (28 slabs total).

### CONCLUSION

This research and its results illustrate the inherent complexity in ensuring high-quality decentralized sanitation solutions and how one component, the user interface, if constructed poorly, can affect the rest of the value chain. The surface appearance did not necessarily indicate that the product met the quality standards, and a wide variability in the quality of sellers' products were found. Although this work was a small study that cannot support general causal claims, precast latrine slabs being sold from private sanitation service providers are generally failing to meet quality standards. Common construction flaws included nonstandard reinforcement materials and spacing, and slabs that are not thick enough or large enough in diameter. Furthermore, dome slabs are essentially round flat slabs with no steel reinforcement bars.

Ensuring adequate quality control of precast latrine slabs is complex, as a degree of trust is involved in concrete work. The production of slabs onsite may ensure that construction is observed and can eliminate transportation costs, but the onus of ensuring the quality of slabs poured onsite would mostly fall on low-income household customers educated by resources such as Harvey (2007) and WEDC (2012), which may not be practical. To reduce monitoring costs, the use of nondestructive testing methods such as the rebound hammer together with measuring slab dimensions are a practical solution for rapidly checking quality standards that could be set by the national government.

Despite the negative results of this small study, the fact that latrine slabs are on the local market indicates that someone is buying them. Each seller has a reputation to maintain and would be motivated to improve their quality control if they lost sales as a consequence of low quality. However, how can a seller compete if they follow quality standards that would raise the cost of making a slab above the price of their competitors? In addition, there are no published reports of local accidents occurring while using or emptying latrines with precast slabs versus those made onsite.

As the need for fecal sludge to be treated offsite increases, prescriptive specifications are even more important. National guidelines based on a local context and proper strategies should be implemented to monitor and evaluate precast latrine slabs for their safety during emptying operations and by users. The findings from this study can be used to inform training and initiatives that engage artisans and suppliers that play a role in the provision of onsite sanitation service delivery.

## References

[cit0001] Centre for Affordable Water and Sanitation Technology (CAWST) 2014 Latrine Construction Manual. CAWST, Calgary, Canada.

[cit0002] Chipeta W. C., Holm R. H., Kamanula J. F., Mtonga W. E. & de los Reyes F. L. 2017 Designing local solutions for emptying pit latrines in low-income urban settlements (Malawi). Physics and Chemistry of the Earth 100, 336-342. DOI:10.1016/j.pce.2017.02.012. 29033689 PMC5625478

[cit0003] Chiposa R., Holm R. H., Munthali C., Chidya R. C. G. & de los Reyes III F. L. 2017 Characterization of pit latrines to support the design and selection of emptying tools in peri-urban Mzuzu, Malawi. Journal of Water Sanitation and Hygiene for Development 7 (1), 151-155. DOI:10.2166/washdev.2017.096.

[cit0004] Harvey P. A. 2007 Excreta Disposal in Emergencies: A Field Manual. Water, Engineering and Development Centre, Loughborough University, Loughborough, UK.

[cit0005] Holm R. H., Kamangira A., Tembo M., Kasulo V., Kandaya H., Gijs Van Enk P. & Velzeboer A. 2018 Sanitation service delivery in smaller urban areas (Mzuzu and Karonga, Malawi). Environment & Urbanization. DOI:10.1177/0956247818766495.

[cit0006] Malawi Government 2008 National Sanitation Policy. Ministry of Irrigation and Water Development, Ministry of Irrigation and Water Development, Lilongwe, Malawi.

[cit0007] Malhotra V. M. & Carino N. J. (eds) 2004 Handbook on Nondestructive Testing of Concrete, 2nd edn. CRC Press, Boca Raton, FL, USA.

[cit0008] National Statistical Office (NSO) (Malawi) & ICF 2017 Malawi Demographic and Health Survey 2015-16. and Rockville, MD, USA. NSO and ICF, Zomba, Malawi.

[cit0009] Rahman N. A. A., Ahmad S. & Zainordin Z. M. 2013 Perception and awareness of leaking for toilet in pre-cast concrete structure. Procedia - Social and Behavioral Sciences 8, 61-69. DOI:10.1016/j.sbspro.2013.08.338.

[cit0010] Szilágyi K. & Borosnyoi A. 2009 50 years of experience with the Schmidt rebound hammer. Concrete Structures 10,46-56.

[cit0011] Walpole S. C., Prieto-Merino D., Edwards P., Cleland J., Stevens G. & Roberts I. 2012 The weight of nations: an estimation of adult human biomass. BMC Public Health 12, 439 DOI:10.1186/1471-2458-12-439. 22709383 PMC3408371

[cit0012] Water, Engineering and Development Centre 2012 Latrine Slabs: an Engineer's Guide. Loughborough University, Loughborough, UK.

[cit0013] Zambia Government 1994 Public Health (Drainage and Latrine) Regulations. Lusaka, Zambia.

